# Stress fracture of the midshaft clavicle associated with sternocostoclavicular hyperostosis—Case report

**DOI:** 10.1016/j.ijscr.2019.03.059

**Published:** 2019-04-19

**Authors:** Shuichi Miyamoto, Makoto Otsuka, Fumio Hasue, Takayuki Fujiyoshi, Koushirou Kamiya, Hitoshi Kiuchi, Tadashi Tanaka, Junichi Nakamura, Sumihisa Orita, Seiji Ohtori

**Affiliations:** a Kimitsu Central Hospital, 1010 Sakurai, Kisarazu City, Chiba, 292-8535, Japan; b Graduate School of Medicine, Chiba University, 1-8-1 Inohana, Chuo-ku, Chiba City, Chiba, 260-8677, Japan; c Center for Advanced Joint Function and Reconstructive Spine Surgery Graduate school of Medicine, Chiba University 1-8-1 Inohana, Chuo-ku, Chiba, 260-8670, Japan

**Keywords:** Stress fracture, Midshaft of the clavicle, Sternocostoclavicular hyperostosis, SAPHO syndrome, Ankylosing spondylitis

## Abstract

**Introduction:**

Stress fracture is generally a result of cumulative and repetitive stress in athletes, which accelerates the normal remodeling process of bones, and the most frequently involved areas are the tibia and metatarsal bones. Therefore, stress fractures of the midshaft of the clavicle are very rare.

**Presentation of case:**

A 58-year-old female was admitted to our hospital because of pain in the middle of the right clavicle. Based on laboratory and radiographic inspection, it was concluded that the stress fracture of the midshaft of the clavicle in this case was caused by sternocostoclavicular hyperostosis (SCCH). Because the clavicular fracture had no displacement or callus formation, conservative treatment with a clavicle band was undertaken. Shoulder function at the final follow-up visit was satisfactory.

**Discussion:**

SCCH is a rare chronic inflammatory disorder of the axial skeleton and ossifying diathesis associated with a predominantly osteogenic response. Synovitis, acne, pustulosis, hyperostosis, and osteitis (SAPHO) syndrome and ankylosing spondylitis (AS) should be considered in the differential diagnosis of SCCH. If a patient with this type of fracture has no history of traumatic injury or sports activity, the differential diagnosis might be very difficult.

**Conclusion:**

We report the case of a female who had a stress fracture of the midshaft of the clavicle associated with SCCH in SAPHO or AS. Although the patient was treated conservatively, and the shoulder function was satisfactory at the final follow-up visit, re-fracture may occur in the future.

## Highlights

• Stress fractures of the midshaft of the clavicle caused by sternocostoclavicular hyperostosis are very rare.
• The differential diagnosis of sternocostoclavicular hyperostosis might be difficult.
• Sternocostoclavicular hyperostosis in the patient was suspected of having either SAPHO syndrome or ankylosing spondylitis.
• The patient was treated conservatively and the shoulder function was satisfactory at the final follow-up.

## 1 Introduction

Stress fracture is generally a result of cumulative and repetitive stress in athletes, which accelerates the normal remodeling process of bones, leading to a weakening of the bone’s outer surface and an increased susceptibility to repeated stress to a localized area [1]. It is accepted that the tibia and metatarsal bones are the most common sites [2], whereas the midshaft clavicle is rarely involved. Previous reports described sports activities, including gymnastics, cricket (fast bowlers), and sternocostoclavicular hyperostosis (SCCH) as triggering factors [[3], [4], [5]].

We report a case of a stress fracture of the midshaft of the clavicle associated with sternocostoclavicular hyperostosis (SCCH) in a female patient suspected of having either synovitis, acne, pustulosis, hyperostosis, and osteitis (SAPHO) syndrome or ankylosing spondylitis (AS). This work is reported consistent with the surgical case report (SCARE) criteria [6].

## 2 Case presentation

Informed consent to allow publication was obtained from the patient who is described in this report. A 58-year-old female visited our hospital because of pain in the middle of the right clavicle. She was able to continue her activities of daily living, mainly housework. There were no episodes of traumatic injury and no sports activity. Her father was diagnosed with rheumatoid arthritis. Physical examination revealed acceptable shoulder function: the patient was able to slowly and passively flex and extend. The shoulder joint range of motion was 140° of forward elevation, 60° of external rotation and 60° of internal rotation with the arm at the side, and 120° of abduction. Although she had clavicular pain related to hyperflexion, hyperextension and hyperabduction, the pain had already peaked when she visited the hospital. There was no arthritis, gastrointestinal disorder, eye disease, morning stiffness, or skin disease. For more than 10 years, she had experienced slight low back pain with activities of daily living, but she had no radiculopathy. On the Schober test, she achieved a distance of only 3 cm with lumbar flexion, and she had a difference in chest measurement of 2 cm between maximum expiration and maximum inspiration at the level of the fourth intercostal space.

**Fig. 1:**
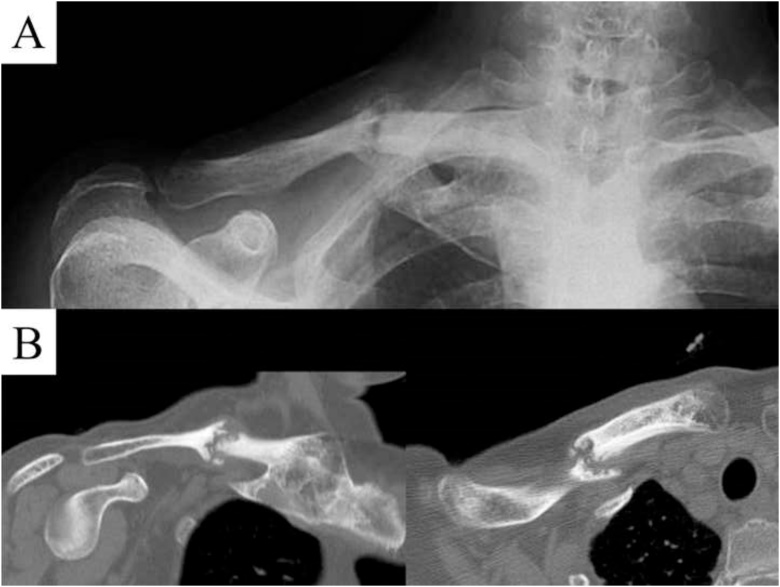
(A) Anteroposterior X-ray image of the clavicle at the time of admission. (B) Sagittal and axial computed tomography of the clavicle.

**Fig. 2:**
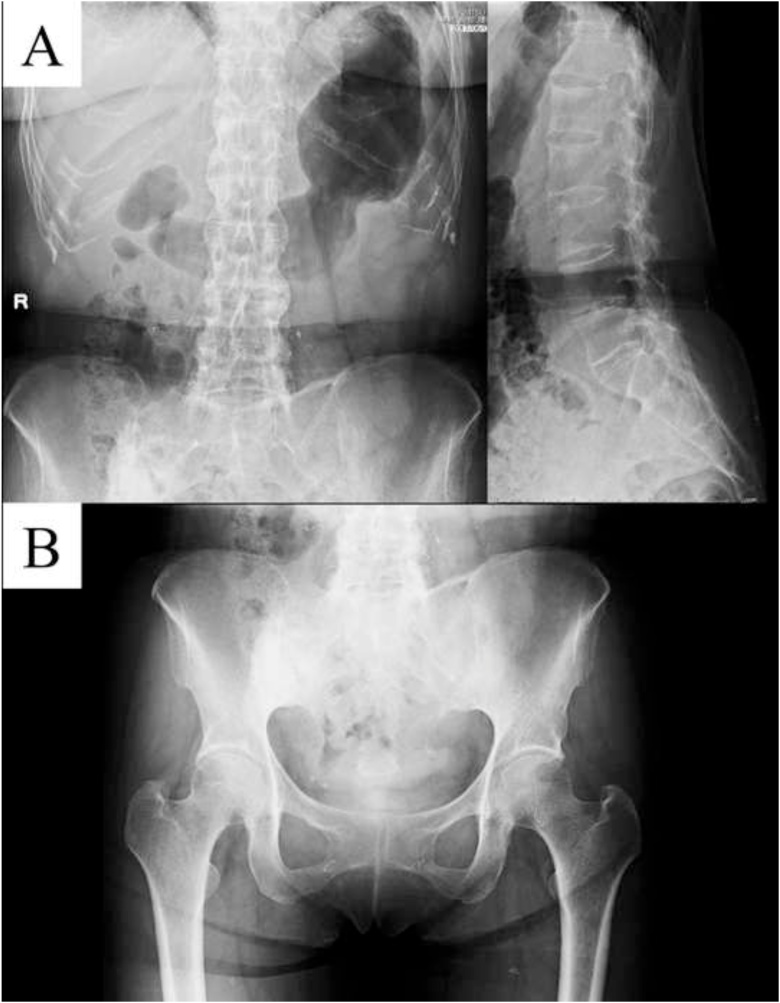
(A) Anteroposterior and lateral X-ray image of the lumbar spine, and (B) anteroposterior X-ray image of the pelvis.

**Fig. 3:**
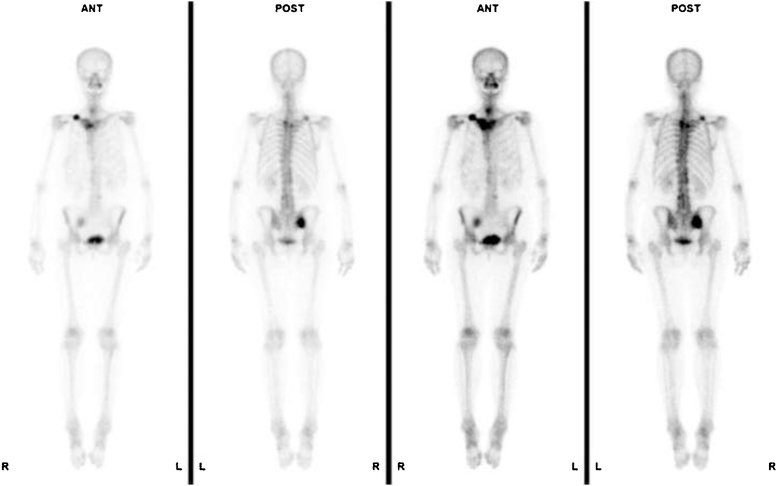
Bone scintigraphy. Anterior and posterior view of the whole body obtained before and after intravenous injection of ^99m^Tc-methylene diphosphonate.

**Fig. 4:**
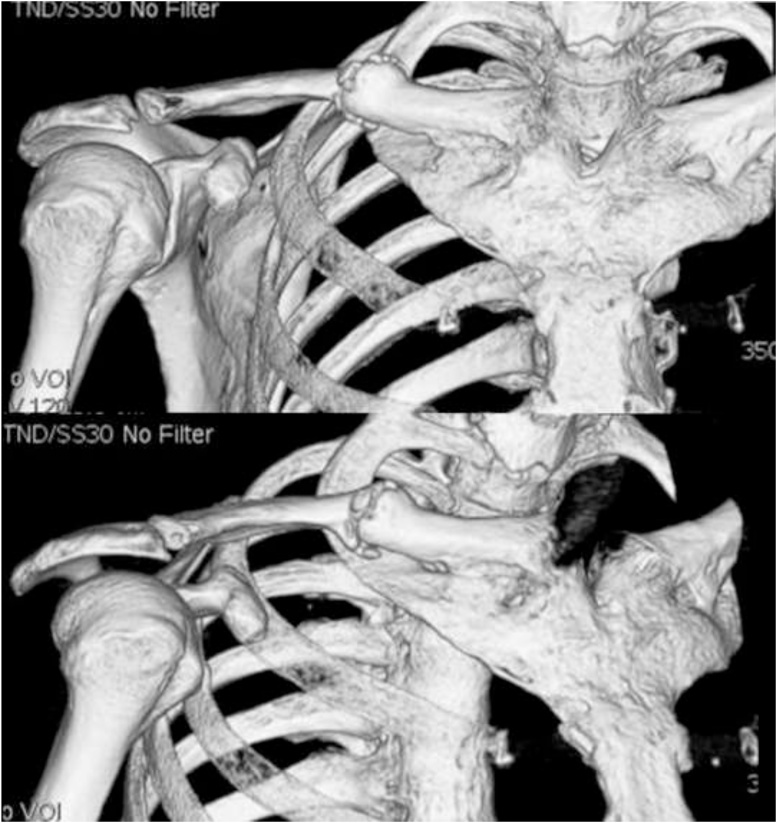
Anteroposterior three-dimensional computed tomography of the clavicle included the sternoclavicular joints.

The relevant abnormal laboratory data at the time of admission was a slightly elevated C-reactive protein (Table1), which was negative at the next admission. The radiograph revealed a fracture of the midshaft of the clavicle with no displacement or callus formation (Fig. 1A). Computed tomography (CT) confirmed the delayed union of the midshaft clavicular fracture with sclerotic, dull margins and ossification of the sternoclavicular joints (SCJ) and first costochondral junctions (Fig. 1B). The radiograph of the lumbar spine and pelvis revealed that the lumbar spine had the characteristics of a bamboo spine with ankylosing changes (Fig. 2A), and the sacroiliac joints had grade 3 bilateral ankylosis (Fig. 2B), as defined by the modified New York radiological sacroiliitis criteria [7]. Bone scintigraphy demonstrated an increasing uptake at the fracture lesion of the midshaft of the clavicle, sternoclavicular joint, sternal manubrium and body, and the right sacroiliac joint (Fig. 3). A CT with three-dimensional reformatted images demonstrated hyperostosis of the bilateral SCJ (Fig. 4). The costoclavicular ligament was ossified and bridged between the proximal clavicle and the first costal cartilage, and the anterior sternoclavicular ligament was ossified and bridged between the clavicle and the sternal manubrium.

**Table 1:** Laboratory findings on admission.

Blood cell count	
White blood cells	5200 /μl
Neutrophils	65.6 %
Lymphocytes	26.7 %
Monocytes	6.4 %
Eosinophils	0.7 %
Basophils	0.6 %
Red blood cells	4.31 × 10^6^
Hemoglobin	11.5 g/dl
Hematocrit	35.40 %
Platelet	243 × 10^3^

Serological test	
C-reactive protein	0.43 mg/dl
HbA1c-NGSP	5.80 %
Rheumatoid factor	2.0 U/ml
HLA-B27	(-)
Anti-cyclic citrullinated peptide antibody	<0.6 U/ml

Serum and urine immunoelectrophoresis	
M-protein	(-)
Bence-Jones protein	(-)

Biochemical test	
Total protein	8 g/dl
Albumin	4.2 g/dl
Aspartate aminotransferase	22 IU/l
Alanine transferase	12 IU/l
Alkaline phosphatase	231 IU/l
Blood urea nitrogen	12.8 mg/dl
Creatinine	0.54 mg/dl
Uric acid	4 mg/dl
Na	139 mg/dl
K	4.5 mg/dl
Cl	103 mg/dl
Ca	9.4 mg/dl

Urinalysis	
Protein	(-)
Nitrites	(-)
Ketones	(-)
Red blood cells	<1 /HPF
White blood cells	1-4 /HPF

**Fig. 5:**
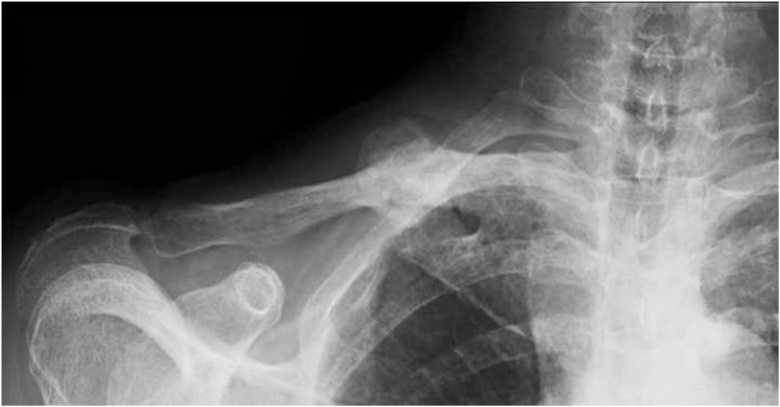
Anteroposterior X-ray image of the clavicle taken 10 months after the first admission.

The patient underwent conservative treatment with a clavicle band for 6 weeks. Active range of motion that avoided overhead movements was permitted as long as pain was within manageable bounds. At the final follow-up visit 10 months after the first admission, consolidation was considered to be achieved with an increasing bridging callus and the disappearance of the fracture line on radiographs (Fig. 5). The shoulder joint range of motion at the final follow-up was 165° of forward elevation, 70° of external rotation with the arm at the side, 160° of abduction, and internal rotation was to the thumb level with the thoracic 6th vertebra. At the final follow-up visit, the patient had only slight pain after intense and long duration of work,　but no problems in activities of daily living or mobility. There was no instability during active or passive movement of the shoulder joint.

## 3 Discussion

In the current case report, we present a stress fracture of the midshaft of the clavicle. This fracture is very rare; it was caused by or resulted from SCCH in SAPHO syndrome or AS. If the patient with a stress fracture of the midshaft of the clavicle has no history of traumatic injury or sports activity, the differential diagnosis might be very difficult.

The sternocostoclavicular joint (SCJ) is the only bony articulation between the arm and the trunk, and most of the scapulothoracic movement occurs though the SCJ. The SCJ consists mainly of a tough intra-articular fibrocartilaginous disc, and costoclavicular, interclavicular and capsular ligaments. The intra-articular fibrocartilaginous disc is attached to the anterior and posterior sternoclavicular ligaments and capsule. Therefore, the SCJ is remarkably stable during normal movement, mainly because of the strength of these ligaments [8]. If the sternocostoclavicular joint were affected by ankylosis with no movement due to SCCH, all stress during upper extremity motion would be increased and concentrated at the midshaft of the clavicle close to the SCCH. As a result, a stress fracture can occur during activities of daily living with no significant trauma, as in this case. Furthermore, the displacement of the fracture on radiography and CT might not be detected. SCCH is a rare chronic inflammatory disorder of the axial skeleton and ossifying diathesis associated with a predominantly osteogenic response [9]. At presentation, the age of the majority of patients was between 30 and 70 years [9,10], and their sex was predominantly female [[9], [10], [11]]. Recently, in a Dutch cohort of 70 patients, a high prevalence of autoimmune disease in patients with adult-onset SCCH was reported [11]. Although SCCH is very rare, osteomyelitis of the clavicle, SAPHO syndrome and AS should be considered in the differential diagnosis of SCCH.

Osteomyelitis was excluded in this patient for two reasons. First, the lytic lesion observed on radiographs and CT images caused by the formation of a sequestrum was not detected. Second, the C-reactive protein was negative without antibiotic treatment at the time of the next admission. The SAPHO syndrome was reported first by a group of French researchers [12] and was based mainly on clinical signs: synovitis, acne, pustulosis, hyperostosis, and osteitis. The diagnosis described by Benhamou et al depended upon the presence of at least one of the inclusion criteria and none of the exclusion criteria [13]. However, the SAPHO syndrome has a different clinical picture with osteoarticular manifestations encompassing a wide spectrum [14,15]. It is possible to diagnose one subtype of the SAPHO syndrome covering a broad spectrum, or to do so accidentally at a certain point when cutaneous symptoms disappear, as in our case.

Spondyloarthritis is a group of interrelated diseases, which consist of AS, psoriatic arthritis, arthritis/spondylitis with inflammatory bowel disease and reactive arthritis [16,17]. Based on a physical examination that revealed no evidence of gastrointestinal disorder, eye disease, or skin disease in addition to a negative HLA-B27, these types of arthritis from the differential diagnosis of spondyloarthritis were excluded. Generally, the diagnostic criteria of AS are based on the modified New York criteria and depend on clinical symptoms and radiographic evidence [7]. This case also met both the clinical and radiographic criteria of AS. HLA-B27 is important to the diagnosis. Although HLA-B27 was negative in our patient, in the Japanese Central Bone Marrow Data Center, the incidence has been reported to be as low as 0.4% [18], a rate much lower than rates reported in the United States and Europe (6%–25%) [19,20]. It might be possible that HLA-B27 does not play a role as a diagnostic marker for AS in the Japanese.

Several reports of stress fracture of the clavicle caused by SCCH or sports have shown good clinical and radiographic results following conservative treatment with a clavicular band or restriction of activity [[3], [4], [5]]. In the current report, the clavicular fracture with callus formation was evaluated radiographically and by CT. There was no instability of the fracture site during passive and active mobility, and the clavicular fracture was not displaced; therefore, the fracture was managed conservatively. At the final follow-up visit, bony union was achieved, and shoulder function was satisfactory. However, we should consider the likelihood of re-fracture with increasing activity in the future. If symptoms of osteoarthritis occur and are complicated, an approach to the treatment of the original disease will be needed.

## 4 Conclusion

We report the case of a female who had a stress fracture of the midshaft of the clavicle associated with SCCH in SAPHO syndrome or AS. The stress fracture was considered to be caused by activities of daily living. Although the patient was treated conservatively, and the shoulder function was satisfactory at the final follow-up visit, re-fracture may occur in the future.

## Conflicts of interest

The authors declare no conflicts of interest regarding the publication of this paper.

## Sources of funding

We did not receive any funding or financial support that may be perceived to have biased this report.

## Ethical approval

All procedures performed in other studies involving human participants were in accordance with the ethical standards approval of the institutional review board and with the 1964 Helsinki declaration and its later amendments or comparable ethical standards.

In case report, Ethical approval has been exempted by our institution.

## Consent

Informed consent to allow publication of this case report and accompanying images was obtained from the patient who is described in this report in outpatient consultation room. A copy of the written consent is available for review by the Editor-in-Chief of this journal on request.

## Author contribution

All authors have read and approved the submitted version. Study design: Shuichi Miyamoto, Makoto Otsuka, Tadashi Tanaka, Junichi Nakamura, Sumihisa Orita, Seiji Ohtori. Drafting the protocol: Shuichi Miyamoto, Makoto Otsuka, Tadashi Tanaka. Advice on the analysis: Junichi Nakamura, Sumihisa Orita, Seiji Ohtori. Data collection and figure preparation: Fumio Hasue, Takayuki Fujiyoshi, Koushirou Kamiya, Hitoshi Kiuchi. First draft of the manuscript: Shuichi Miyamoto.

## Registration of research studies

We registered my study and obtained UIN in Research Registry.

Our research registry UIN was 4637.

## Guarantor

Shuichi Miyamoto and Seiji Ohtori.

## Provenance and peer review

Not commissioned, externally peer-reviewed.
